# Up-front mutation detection in circulating tumor DNA by droplet digital PCR has added diagnostic value in lung cancer

**DOI:** 10.1016/j.tranon.2022.101589

**Published:** 2022-11-19

**Authors:** Esther Visser, Remco de Kock, Sylvia Genet, Ben van den Borne, Maggy Youssef-El Soud, Huub Belderbos, Gerben Stege, Marleen de Saegher, Susan van ’t Westeinde, Maarten Broeren, Federica Eduati, Birgit Deiman, Volkher Scharnhorst

**Affiliations:** a Department of Biomedical Engineering, Eindhoven University of Technology, Eindhoven, the Netherlands; b Catharina Hospital Eindhoven, Eindhoven, the Netherlands; c Máxima Medical Center, Eindhoven, Veldhoven, the Netherlands; d Expert Center Clinical Chemistry Eindhoven, Eindhoven, the Netherlands; e Institute for Complex Molecular Systems, Eindhoven University of Technology, Eindhoven, the Netherlands; f Amphia Hospital, Breda, the Netherlands; g Anna Hospital, Geldrop, the Netherlands; h Sint Jans Gasthuis, Weert, the Netherlands; i Maasstad Hospital, Rotterdam, the Netherlands; j Eindhoven Artificial Intelligence Systems Institute, Eindhoven University of Technology, Eindhoven, the Netherlands

**Keywords:** Liquid biopsy, Droplet-digital PCR, NSCLC, Mutation analysis, Circulating tumor DNA, cfDNA, cell-free DNA, ctDNA, circulating tumor DNA, ddPCR, droplet digital polymerase chain reaction, DNA, deoxyribonucleic acid, NGS, next generation sequencing, NPV, negative predictive value, NSCLC, non-small-cell lung cancer, PPV, positive predictive value, SCLC, small-cell lung cancer, tDNA, tumor DNA

## Abstract

Identification of actionable mutations in advanced stage non-squamous non-small-cell lung cancer (NSCLC) patients is recommended by guidelines as it enables treatment with targeted therapies. In current practice, mutations are identified by next-generation sequencing of tumor DNA (tDNA-NGS), which requires tissue biopsies of sufficient quality. Alternatively, circulating tumor DNA (ctDNA) could be used for mutation analysis. This prospective, multicenter study establishes the diagnostic value of ctDNA analysis by droplet digital PCR (ctDNA-ddPCR) in patients with primary lung cancer.

CtDNA from 458 primary lung cancer patients was analyzed using a panel of multiplex ddPCRs for *EGFR* (Ex19Del, G719S, L858R, L861Q and S768I)*, KRAS* G12/G13 and *BRAF* V600 mutations. For 142 of 175 advanced stage non-squamous NSCLC patients tDNA-NGS results were available to compare to ctDNA-ddPCR. tDNA-NGS identified 98 mutations, of which ctDNA-ddPCR found 53 mutations (54%), including 32 of 45 (71%) targetable driver mutations. In 2 of these 142 patients, a mutation was found by ctDNA-ddPCR only. In 33 advanced stage patients lacking tDNA-NGS results, ctDNA-ddPCR detected 15 additional mutations, of which 7 targetable.

Overall, ctDNA-ddPCR detected 70 mutations and tDNA-NGS 98 mutations in 175 advanced NSCLC patients. Using an up-front ctDNA-ddPCR strategy, followed by tDNA-NGS only if ctDNA-ddPCR analysis is negative, increases the number of mutations found from 98 to 115 (17%). At the same time, up-front ctDNA-ddPCR reduces tDNA-NGS analyses by 40%, decreasing the need to perform (additional) biopsies.

## Highlights

• ctDNA-ddPCR identified 54% of all and 71% of targetable mutations in advanced NSCLC with tDNA-NGS as gold standard.
• In 17 of 175 patients, mutations were solely identified by ctDNA-ddPCR.
• Up-front ctDNA-ddPCR could be performed prior to tDNA-NGS.
• Only patients without a mutation in ctDNA-ddPCR analysis need tDNA-NGS.

## Introduction

Introduction of targeted therapy has improved the prognosis of patients with advanced stage non-squamous non-small-cell lung cancer (NSCLC) with an actionable driver mutation [1,2]. Targeted therapies include inhibition of signaling pathways that promote proliferation and survival of tumor cells by blocking receptors or protein kinases harboring these mutations [1]. Currently, several therapies targeting driver mutations in e.g. *EGFR, BRAF* and *ALK-ROS1* genes are used in clinical practice, that lead to longer progression free survival of advanced stage NSCLC patients compared to chemotherapy [2].

To identify patients that may benefit from targeted therapies, guidelines recommend to test gene alterations for all patients with advanced or metastatic non-squamous NSCLC [2], [3], [4], [5], [6]. In current practice, molecular aberrations are detected in tissue-derived tumor DNA (tDNA) by next generation sequencing (NGS) or fluorescent *in situ* hybridization (FISH) [6,7]. However, tumor tissue cannot always be obtained via biopsies, for example due to inaccessibility of the tumor or due to poor condition of the patient [8], [9], [10]. Moreover, tissue samples may contain insufficient tumor cells for molecular analysis, requiring additional biopsies [[7], [8], [9],^11^,^12^].

Alternatively, the detection of driver mutations could be performed on circulating tumor DNA (ctDNA) derived from liquid biopsies, for example from plasma [[8], [9], [10],^12^]. Liquid biopsies are a minimally invasive way to obtain ctDNA, not only from the primary tumor but potentially also from metastatic sites [8,10]. Thus, analysis of ctDNA may better cover the heterogeneity of the tumor [8,10]. Recently, droplet digital PCR (ddPCR) was shown to be a fast and sensitive method for detection of mutations in ctDNA [13,14]. Using multiplex ddPCR, several mutations can be analyzed in parallel in one reaction, which reduces the amount of ctDNA needed and laboratory costs [14], [15], [16].

This study aimed to establish the value of ctDNA-ddPCR mutation analysis during the diagnostic phase of lung cancer. To that end, ctDNA-ddPCR analysis was performed in plasma of all patients suspected of primary lung cancer. For advanced stage non-squamous NSCLC patients who routinely undergo tDNA-NGS mutation analysis, the diagnostic yield of ctDNA-ddPCR analysis was compared to routine practice.

## Materials and methods

### Subjects and study design

This study is part of a prospective, multicenter clinical trial, the lung marker study, that has been approved by the Medical Research Ethics Committees United (NL9146). 788 patients suspected of lung cancer were included by their lung physician in six hospitals in the Netherlands between June 2017 and May 2021. After obtaining written informed consent, blood samples were collected during the diagnostic phase. The plasma samples of all patients were analyzed by ctDNA-ddPCR to identify driver mutations in *EGFR, KRAS* and *BRAF,* which are frequently occurring mutations in NSCLC patients [1].

**Fig. 1:**
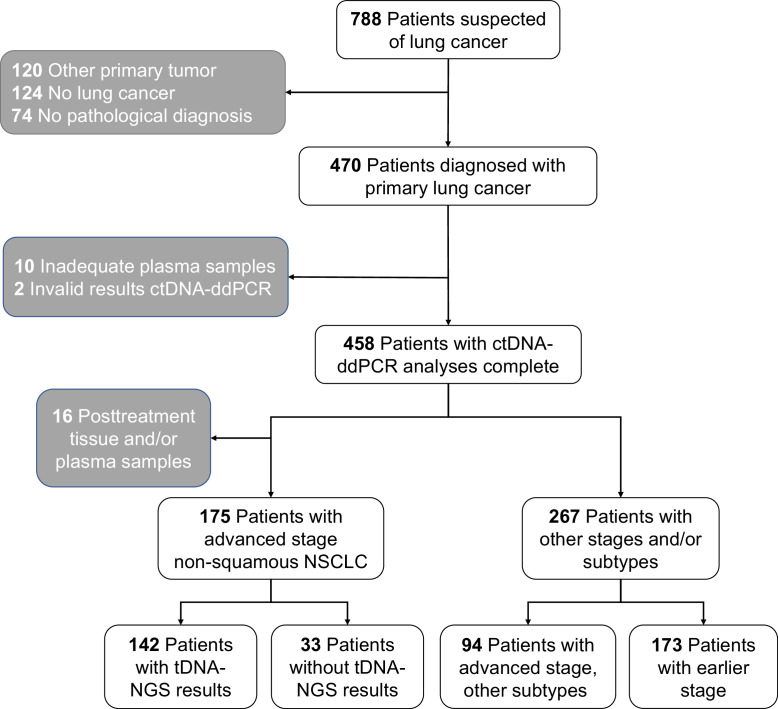
Flowchart of inclusion of patients and division of subgroups for analysis. NSCLC: non-small-cell lung cancer; tDNA-NGS: tumor DNA - Next Generation Sequencing; ctDNA-ddPCR: circulating tumor DNA – droplet digital PCR.

Patients without a pathologically confirmed primary lung cancer were excluded from analyses. Patients with other primary tumors were excluded, since the ctDNA could originate from these tumors as well. In total, 318 patients were excluded and 458 patients were available for analyses (Fig. 1). Diagnosis and staging of the primary lung cancer patients was done according to Dutch Guidelines [7,17].

For patients with advanced stage non-squamous NSCLC, molecular aberrations in tissue-derived tDNA were retrieved from clinical pathology reports in the patients’ electronic health records. Adequate tissue samples should consist of sufficient tumor cells (>10%, variant allele frequency >5%) or sufficient DNA (> 10 ng) [7]. The analyses of molecular aberrations in tDNA, referred to as tDNA-NGS, were performed by NGS (Ion Torrent, sensitivity 5–10% mutant allele, Qiagen Genereader, sensitivity 10% mutant allele or Illumina Ampliseq followed by sequencing by synthesis, sensitivity 5% mutant allele) and translocations and amplifications by FISH. The tDNA-NGS analyses were performed in different centers and were also updated during the study timeframe, resulting in differences between the panels used. Following guidelines, these panels covered at least *EGFR, KRAS, ALK, ROS1, BRAF, RET, HER2* and starting in 2020 also *MET, NTRK1/2/3* and *NRG* [7,18]. Various non-actionable alterations were also analyzed by tDNA-NGS, varying per patient and depending on the center and time of analysis.

To investigate the diagnostic performance of mutation analysis by ctDNA-ddPCR in advanced stage non-squamous NSCLC patients, its results were compared to current molecular analyses. Therefore, tDNA-NGS mutation analysis, including all actionable and non-actionable alterations, was used as a reference, if available. Only data from plasma and tissue samples obtained before start of treatment were considered. The median time between venipuncture for ctDNA-ddPCR analysis and tissue sampling for tDNA-NGS analysis was 9 days (5–16 days), with 47.9% of the plasma samples obtained before tissue samples, 45.1% after and 7.0% on the same day. The performance of ctDNA-ddPCR was compared per detected mutation and per patient. For patients with multiple mutations detected by tDNA-NGS, identification of at least one of the mutations by ctDNA-ddPCR was counted as ‘detected’. Molecular aberrations in *EGFR, BRAF* V600*, KRAS* G12C*, MET* (exon 14 skipping and amplification) and *ALK* were considered clinically targetable.

### Sample collection, processing and ddPCR mutation analysis

Whole blood samples were collected and processed to obtain plasma and cell-free DNA (cfDNA) as previously described [16]. The presence of mutations in *KRAS* G12/G13*,* covering the point mutations G12A, G12C, G12D, G12R, G12S, G12V and G13D was determined using the ddPCR *KRAS* G12/G13 Screening Multiplex Kit (QX200 ddPCR System, Bio-Rad Laboratories, Hercules, CA). In cases with a *KRAS* mutation, presence of the *KRAS* G12C mutation was investigated. The *BRAF* V600 mutations V600E, V600K and V600R were analyzed by using the ddPCR *BRAF* V600 Screening Kit (Bio-Rad Laboratories). Additionally, presence of the *EGFR* mutations Ex19Del, G719S, L858R, L861Q and S768I was determined using a pentaplex reaction as previously described [15]. The cfDNA concentrations of wildtype and mutant *KRAS* with 95% Poisson-based CI were calculated using the fraction of positive and negative droplets [16]. The sum of concentrations of wildtype and mutant *KRAS* were used as a measure for the concentrations of cfDNA, expressed as copies per milliliter plasma [16].

### Statistical analysis

The sensitivity, specificity, positive predictive value (PPV) and negative predictive value (NPV) of ctDNA-ddPCR analysis were evaluated on patient level taking tDNA-NGS analysis as reference. To determine whether the detection of the mutations by ctDNA-ddPCR could have been influenced by the concentrations of cfDNA, two sided Mann-Whitney-U tests were used, considering *p* < 0.05 as significantly different. Results are presented as numbers with frequencies (%) or median with interquartile range (25th–75th percentile). Analyses were performed using the Python package SciPy (Python version 3.8.5, Scipy version 1.7.1).

## Results

### Study cohort

Of the 458 primary lung cancer patients, 175 patients had advanced stage non-squamous cell NSCLC with a pre-treatment tissue and plasma sample and, following the guidelines, tDNA-NGS was attempted (Fig. 1). tDNA-NGS was successfully performed for 142 patients (81.1%). The majority of these patients were stage IV patients (92.3%) and 81.0% was diagnosed with adenocarcinoma (Table 1).

**Table 1:** Characteristics of patients in the different groups.

Patient subgroup	Advanced stage, non-squamous NSCLC	Advanced stage, non-squamous NSCLC	Advanced stage, other subtypes	Earlier stage disease
Mutation analysis	tDNA-NGS + ctDNA-ddPCR	ctDNA-ddPCR	ctDNA-ddPCR	ctDNA-ddPCR
Number of patients	142	33	94	173
Age	67 (59–71)	70 (62–74)	71 (64–76)	69 (63–73)
Sex				
Females	66 (46.5%)	15 (45.5%)	30 (31.9%)	93 (53.8%)
Males	76 (53.5%)	18 (54.5%)	64 (68.1%)	80 (46.2%)
Smoking history				
Active	51 (35.9%)	13 (39.4%)	31 (33.0%)	67 (38.7%)
Former	72 (50.7%)	15 (45.5%)	54 (57.4%)	92 (53.2%)
Never	13 (9.2%)	1 (3.0%)	2 (2.1%)	5 (2.9%)
Unknown	6 (4.2%)	4 (12.1%)	7 (7.4%)	9 (5.2%)
Tumor type				
NSCLC	142 (100.0%)	33 (100.0%)	57 (60.6%)	170 (98.3%)
*Adenocarcinoma*	115 (81.0%)	22 (66.7%)	0 (0.0%)	91 (52.6%)
*Squamous cell carcinoma*	0 (0.0%)	0 (0.0%)	57 (60.6%)	59 (34.1%)
*LCNEC*	6 (4.2%)	2 (6.1%)	0 (0.0%)	7 (4.0%)
*NOS/Unknown*	21 (14.8%)	9 (27.3%)	0 (0.0%)	13 (7.5%)
SCLC	0 (0.0%)	0 (0.0%)	37 (39.4%)	3 (1.7%)
Stages				
0	0 (0.0%)	0 (0.0%)	0 (0.0%)	2 (1.2%)
I	0 (0.0%)	0 (0.0%)	0 (0.0%)	62 (35.8%)
II	0 (0.0%)	0 (0.0%)	0 (0.0%)	47 (27.2%)
IIIa	0 (0.0%)	0 (0.0%)	0 (0.0%)	62 (35.8%)
IIIb/IIIc	11 (7.7%)	12 (36.4%)	25 (26.6%)	0 (0.0%)
IV	131 (92.3%)	21 (63.6%)	69 (73.4%)	0 (0.0%)

For the remaining 33 patients with advanced NSCLC, only ctDNA-ddPCR but no tDNA-NGS results were available, due to insufficient tissue (*n* = 14), patients abstaining from treatment (*n* = 4) or unknown reasons (*n* = 15). Of these patients, 63.6% were stage IV patients and 36.4% stage IIIb/IIIc patients (Table 1).

Additionally, ctDNA-ddPCR results were generated for 267 patients for whom molecular analysis is not recommended by guidelines: 94 patients with advanced lung cancer of squamous cell (*n* = 57) and small-cell (SCLC) type (*n* = 37) and 173 patients diagnosed with stage 0, I, II or IIIa lung cancer of any type (Fig. 1, Table 1). Patient characteristics of the subgroups are shown in Table 1.

### Comparison of mutation analyses by ctDNA-ddPCR and tDNA-NGS

**Fig. 2:**
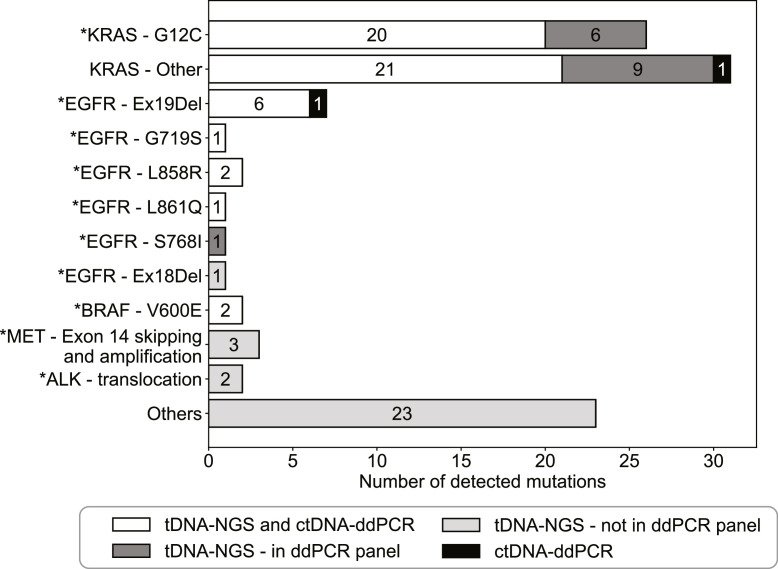
Mutations identified by ctDNA-ddPCR (black), tDNA-NGS (gray) or both (white) in patients with advanced stage non-squamous NSCLC (*n* = 142). For mutations only identified by tDNA-NGS, the presence (dark gray) or absence (light gray) in the ddPCR panel is indicated. The mutations absent in the ddPCR panel were only shown when clinically targetable, otherwise the mutations were indicated as ‘others’. Further specification of the ‘others’ groups can be found in Supplemental Table 1. For the uncommon alteration EGFR - Ex18Del (Glu709_Thr710delinsAsp), some case studies showed potential clinically relevance [19,20]. * Clinically targetable mutations.

To determine the performance of ctDNA-ddPCR mutation analysis in advanced stage non-squamous NSCLC patients, its results were compared to the mutations identified by tDNA-NGS. For 47 out of 142 advanced NSCLC patients molecular alterations were undetectable by both methods. In the remaining 95 patients, 100 mutations were identified (Fig. 2, Supplemental Table 1). Seventy-one (71%) mutations were covered by the panels of both methods and 53 (53%) mutations were actually detected by both ctDNA-ddPCR and tDNA-NGS. In addition, 2 (2%) mutations were detected with ctDNA-ddPCR only and 16 (16%) with tDNA-NGS only. Furthermore, tDNA-NGS identified 29 (29%) mutations that were undetectable by the ddPCR panel used.

For 91 of the 95 mutation-positive patients (96%), a single mutation was detected, whereas for 4 patients (4%) multiple mutations were found (Table 2). In three patients, one of the mutations was identified by both tDNA-NGS and ctDNA-ddPCR and an additional mutation was only identified by tDNA-NGS. None of these additional mutations were relevant for treatment, i.e. actionable.

**Table 2:** Patients with multiple mutations, detected by both tDNA-NGS and ctDNA-ddPCR or tDNA-NGS only.

Patient	Detected by tDNA-NGS and ctDNA-ddPCR	Detected by tDNA-NGS
1	*EGFR –* G719S* ^#^	*EGFR -* S768I* ^#^
2	*EGFR –* Ex19Del* ^#^	*PIK3CA -* E542K
3	*KRAS –* G12D *^#^*	*IDH1 -* exon 4
4		*TP53*
		*TERT* promotor
		*CTNNB1* - N380K

*Clinically targetable mutations; # Mutations available in ddPCR panel.

Forty-six of the 100 detected mutations were clinically targetable and thus valuable for optimal treatment decision. Forty (85%) of these mutations were covered by the panels of ctDNA-ddPCR and tDNA-NGS and 32 (70%) were effectively identified by both methods (Fig. 2, Table 3). One actionable mutation in *EGFR* (Ex19Del) was only found by ctDNA-ddPCR. In addition, 7 mutations were only identified by tDNA-NGS (6 in *KRAS* (G12C) and 1 in *EGFR* (S768I)). The other 6 mutations identified by tDNA-NGS alone were not included in the ddPCR panel.

**Table 3:** Comparison of mutations identified by tDNA-NGS, ctDNA-ddPCR or both methods in patients with advanced-stage non-squamous NSCLC.

	tDNA-NGS + ctDNA-ddPCR	tDNA-NGS	ctDNA-ddPCR
Targetable mutation (*n* = 46)	32 (70%)	13 (28%)	1 (2%)
Non-targetable mutations (*n* = 54)	21 (39%)	32 (59%)	1 (2%)
Total mutations (*n* = 100)	53 (53%)	45 (45%)	2 (2%)

71 of the 100 mutations were covered by the ddPCR panel used, of which 55 (77%) were actually detected, while 16 (23%) were missed, which might have been influenced by cfDNA concentrations (Fig. 2, Supplemental Table 1). The concentration of cfDNA was significantly lower in plasma of patients whose mutation was missed by ctDNA-ddPCR (1788 copies/mL plasma (1554 - 2282)) compared to patients whose mutation was detected by ctDNA-ddPCR (3426 copies mL/plasma (1997 – 6984)) (*p* = 0.007, Supplemental Fig. 1). However, the cfDNA concentrations of plasma samples of both groups overlapped, showing that detection of mutations would be possible for these cfDNA concentrations.

Determining the diagnostic performance of ctDNA-ddPCR per patient with tDNA-NGS as reference, resulted in a sensitivity of 57.0%, specificity of 95.9%, PPV of 96.4% and NPV of 54.0% for all mutations (Supplementary Table 2A). For the clinically targetable mutations, a sensitivity of 71.1%, specificity of 99.0%, PPV of 97.0% and NPV of 88.1% was reached by ctDNA-ddPCR (Supplementary Table 2B). For mutations detectable by the ddPCR panel, ctDNA-ddPCR had a sensitivity of 77.9%, specificity of 97.3%, PPV of 96.4% and NPV of 82.8% (Supplementary Table 2C).

### Mutations identified by ctDNA-ddPCR in patients without tDNA-NGS results

Tissue mutation analysis is recommended for all advanced stage non-squamous NSCLC patients. Table 4 shows that out of 33 advanced NSCLC patients without tDNA-NGS results, 15 (46%) had a mutation by ctDNA-ddPCR and 7 of these mutations were actionable (6 in *KRAS* G12C and 1 in *EGFR* Ex19Del).

**Table 4:** Mutations identified by ctDNA-ddPCR for patients with advanced stage non-squamous cell NSCLC without NGS, advanced stages of other histological subtypes and early stage lung cancer.

	Advanced stage, non-squamous NSCLC	Advanced stage, other subtypes	Earlier stage disease
Mutation analysis	ctDNA-ddPCR	ctDNA-ddPCR	ctDNA-ddPCR
Number of patients (*n* = 267)	33	94	173
Total mutations (*n* = 27)	15 (45.5%)	4 (4.3%)	8 (4.6%)
*KRAS (n* *=* *22)*	14 (42.4%)	1 (1.1%)	7 (4.0%)
*G12C (n* *=* *6)*	6 (18.2%)	–	–
*EGFR (n* *=* *5)*	1 (3.0%)	3 (3.2%)	1 (0.6%)
*L858R (n* *=* *3)*	*0 (0.0%)*	*2 (2.1%)*	*1 (0.6%)*
*Ex19Del (n* *=* *1)*	*1 (3.0%)*	*0 (0.0%)*	*0 (0.0%)*
*S768I (n* *=* *1)*	*0 (0.0%)*	*1 (1.1%)*	*0 (0.0%)*
*BRAF (n* *=* *0)*	0 (0.0%)	0 (0.0%)	0 (0.0%)

Overall, in 175 advanced stage non-squamous NSCLC patients, ctDNA-ddPCR found 70 (40%) mutations, including 40 actionable mutations (22.9%); tDNA-NGS detected 98 molecular alterations in 93 patients (53.1%), of whom 45 patients with actionable mutations (25.7%). By combining the results of both methods, 115 mutations were detected in 110 (62.9%) patients, 53 (30.3%) of whom with actionable mutations.

Advanced stage patients of other tumor etiologies and early stage patients are currently not eligible for tDNA analysis. As this study performed ctDNA-ddPCR analysis for all patient groups, mutation analyses are also available for patients not recommended for molecular analysis by guidelines (Table 4). In a group of patients with advanced stage lung cancer other than non-squamous NSCLC (*n* = 94), driver mutations were detected in 4 patients (4.3%). One mutation was detected in *KRAS* (G12/G13) and 3 in *EGFR* (2 L858R, 1 S768I) for three patients with squamous cell carcinoma with a smoking history, and one patient with SCLC.

Also, in a group of early stage primary lung cancer patients with potentially resectable tumors (stage I/II/IIIa, *n* = 173), 7 *KRAS* (G12/G13) mutations and 1 *EGFR* L858R mutation were detected (Table 4). These mutations were identified in six patients with adenocarcinoma (four stage IIa and two stage IIIa patients), one patient with LCNEC stage IIb and one patient with stage IIb squamous cell carcinoma. For early stage non-squamous NSCLC patients, cfDNA concentrations were significantly lower (1575 (1050 – 2719) copies/mL plasma)) than for advanced stage non-squamous NSCLC patients (2903 (1709–6622) copies/mL plasma) (*p* = 1.8 × 10^−7^), although the concentration ranges still overlapped (Supplemental Fig. 2). Moreover, mutations could be detected by ctDNA-ddPCR in advanced stage patients with low cfDNA concentrations, suggesting that the cfDNA concentrations may not limit the detection of mutations in early stage patients.

## Discussion

This study evaluated the added value of ddPCR mutation analysis on plasma-derived ctDNA for primary lung cancer patients compared to current clinical practice. Advanced stage non-squamous NSCLC patients are recommended to have mutation analyses and we compared the performance of ctDNA-ddPCR to standard care, i.e. molecular analysis of tissue-derived tDNA. Moreover, we described the mutations identified by ctDNA-ddPCR in patient groups for whom tDNA-NGS is recommended but was unavailable or not recommended.

53 of the 55 mutations identified by ctDNA-ddPCR were also detected by tDNA-NGS, which leads to a specificity of 95.9% and a PPV of 96.4% taking tissue tDNA-NGS as gold standard. Previous studies also showed that mutations could be detected in ctDNA with a high PPV and/or specificity and therefore a low number of false-positive results [9,21,22]. In our study, two additional mutations were identified by ctDNA-ddPCR compared to tDNA-NGS. For one patient an *EGFR* Ex19Del mutation was detected with ctDNA-ddPCR. Tissue re-biopsy was performed and by tDNA-NGS the presence of this mutation was confirmed. Possibly due to tumor heterogeneity, the mutation was missed in the tDNA of the first tissue-biopsy. Previously it was described that ctDNA may better represent tumor heterogeneity and potentially also metastases [2,10,23]. For the other patient, a *KRAS* mutation was identified by ctDNA-ddPCR only, but, since this mutation was not targetable, re-biopsy was not performed.

Due to the more extensive coverage of the tissue mutation analysis, 29 mutations were detected by tDNA-NGS only. These mutations were missing in our ddPCR panel since it was developed to cover targetable and commonly occurring alterations in lung cancer, whereas NGS also covers less common alterations. Therefore, targetable alterations such as *MET, ALK, ROS, RET, HER2, NTRK* and *NRG* were only available in the tissue mutation analysis. Since guidelines recommend testing for these alterations, the current panel of ddPCRs cannot replace mutation analysis on tumor tissue. Future research could therefore focus on extension of the ddPCR panel or application of other methods such as ctDNA-NGS analysis to cover more relevant mutations [9,14].

In addition, ctDNA-ddPCR missed 16 technically detectable mutations, possibly due to a concentration of ctDNA in plasma or DNA extract that is below the limit of detection [9,10,14]. For the total cfDNA concentrations, we found an overlap between the concentrations of patients with undetected mutations compared to patients with detected mutations. Therefore, the ctDNA-ddPCR analyses were potentially not limited by the cfDNA concentrations, but by other factors that may influence the fractional abundance of mutant ctDNA in cfDNA. The sensitivity challenge of mutation detection in plasma ctDNA compared to tissue tumor was also described by previous studies, as similar false-negative rates were found [9,11,24].

Single mutations were detected in the majority of the advanced stage non-squamous NSCLC patients in our population with successful tumor DNA analyses, but in a small subset of patients (2.8%) multiple mutations were detected. Sholl et al. [25], investigating a larger population of 1007 stage IV or recurrent lung-adenocarcinoma patients, identified multiple mutations in 27 samples (2.7%), which is comparable to our results. Due to the limited panel of targets in ctDNA-ddPCR analyses, it could occur that a clinically non-relevant mutation is detected in plasma by ctDNA-ddPCR, and another, treatable mutation is missed [25]. However, combining mutation data from Sholl et al. [25] with our PCR panel shows that this situation might lead to a missed targetable mutations in approximately 0.5% of the population only. Even though these missed targetable mutations are a limitation of current ctDNA-ddPCR analyses, targetable mutations could also be missed by tDNA-NGS due to e.g. uninformative biopsies. Since we have shown here that ctDNA-ddPCR identified 4.6% additional targetable mutations, the expected loss of targetable mutations is considerably lower than the gained information.

Additional mutations were identified by ctDNA-ddPCR in 15 advanced stage non-squamous NSCLC patients lacking tDNA-NGS results, e.g. because tissue biopsies could not be retrieved or could not be analyzed due to insufficient amount of tumor cells. For one patient, a targetable *EGFR* Ex19Del was detected in plasma-derived ctDNA, while multiple samples of pleural fluid did not contain enough tumor cells for tissue mutation analyses. This patient was treated with the TKI Osimertinib, partial response as best response and progression of disease after 6 months. The other 14 patients had *KRAS* G12/G13 mutations detected in ctDNA, including 6 with potentially targetable *KRAS* G12C mutations. None of the patients with a G12C mutation were treated with Sotorasib, since this drugs has only recently been approved as second line therapy in the Netherlands. Overall, for patients with advanced stage non-squamous NSCLC in this study (*n* = 175), the total detected mutations increased from 98 (tDNA-NGS alone) to 115 (17%) by combining tDNA-NGS and ctDNA-ddPCR. Therefore, besides confirming part of the mutations that were also found by tDNA-NGS, ctDNA-ddPCR increased the number of mutations compared to current clinical practice, i.e. tDNA analysis.

In conclusion, for advanced stage non-squamous NSCLC patients, mutations detected by ddPCR in plasma-derived ctDNA agree well with tumor-derived tDNA-NGS findings (PPV = 96.4%). The presence of mutations in the tumor cannot be excluded in case ctDNA-ddPCR fails to detect a mutation, as the NPV of ctDNA-ddPCR is 54.0% only. Adding ctDNA-ddPCR analysis to tDNA-NGS, increases the detected mutations by 17%.

As ctDNA-ddPCR is marginally invasive, less complex, relatively cheap and can be performed in a single day, a potential clinical workflow could be to first screen advanced stage non-squamous NSCLC patients for the presence of mutations by performing up-front ctDNA-ddPCR analysis. Only if ctDNA-ddPCR does not show mutations, further molecular analysis of tDNA by NGS needs to be performed. Even though tissue biopsies are necessary for diagnosis of the tumor, repeated biopsies that are performed only for molecular analyses could potentially be saved by using this workflow. Time to treatment may thus be shortened. Moreover, targeted therapy can be given to patients for whom tissue biopsies cannot be retrieved, e.g. due to poor condition, and ctDNA analysis shows an actionable driver mutation. Although there would be a small risk to miss targetable co-mutations, this up-front analysis would allow to identify additional targetable mutations compared to tDNA-NGS alone. Another advantage of screening for mutations by ctDNA-ddPCR is that the results can be used as baseline for monitoring of treatment response [26].

This study identified mutations by ctDNA-ddPCR in 4% of the patients with subtypes of advanced-stage lung cancer which fall outside guideline recommendations for molecular analysis. This low detection rate might be as expected, as the incidence of mutations in squamous cell carcinoma patients was described to be lower than in adenocarcinoma patients [2,4]. Furthermore, ctDNA-ddPCR identified mutations in 5% of patients with earlier stage tumors. The sensitivity to detect mutations in earlier stage patients could potentially be limited by the small quantities of ctDNA in plasma [14]. For the overall cfDNA concentrations, we found that lower concentrations of cfDNA in early stage patients compared to advanced stage patients, but mutations could still be detected in advanced stage patients with low cfDNA concentrations. This finding suggests that the difference in detection rate between early and advanced stage patients would be caused by other aspects of tumor biology influencing the fractional abundance of mutant ctDNA in cfDNA. Therefore, for both groups of patients, the relevance of mutation analysis still needs to be proven.

## Conclusions

In summary, this study shows that addition of mutation analysis by ctDNA-ddPCR to tDNA-NGS improves mutation detection in advanced stage non-squamous NSCLC patients. Therefore, introduction of up-front ctDNA-ddPCR analysis into routine molecular work-up may be warranted.

## Funding sources

Supported by 10.13039/100004325AstraZeneca grant AZNL201700295, The Netherlands Organization for Scientific Research (NWO) via LIFT grant 731.017.405 and Catharina Onderzoeksfonds institutional fund grant 2017–02. The funders had no involvement in study design; collection, analysis and interpretation of data; writing of the report and submission of the article.

## CRediT authorship contribution statement

**Esther Visser:** Formal analysis, Investigation, Visualization, Data curation, Software, Writing – original draft. **Remco de Kock:** Investigation, Data curation, Methodology, Writing – review & editing. **Sylvia Genet:** Investigation, Data curation, Writing – review & editing. **Ben van den Borne:** Resources. **Maggy Youssef-El Soud:** Resources, Writing – review & editing. **Huub Belderbos:** Resources. **Gerben Stege:** Resources, Writing – review & editing. **Marleen de Saegher:** Resources. **Susan van ’t Westeinde:** Resources. **Maarten Broeren:** Resources, Writing – review & editing. **Federica Eduati:** Supervision, Writing – review & editing. **Birgit Deiman:** Methodology, Investigation, Writing – review & editing. **Volkher Scharnhorst:** Conceptualization, Project administration, Supervision, Funding acquisition, Writing – review & editing.

## Appendix Supplementary materials

Image, application 1
