# Supplementary material for: Up-front mutation detection in circulating tumor DNA by droplet digital PCR has added diagnostic value in lung cancer
Source: Transl Oncol. 2022 Nov 19;27:101589. doi: 10.1016/j.tranon.2022.101589 (PMC9679361; doi:10.1016/j.tranon.2022.101589)
Supplement: Supplementary file 1 [file mmc1.pdf]

Supplemental Materials

Supplemental Table 1 – Mutations identified in advanced stage non-squamous NSCLC patients (n = 142) by tDNA-NGS, ctDNA-ddPCR or both methods.

	tDNA-NGS + ctDNA-ddPCR	tDNA-NGS	ctDNA-ddPCR
**Total mutations (n = 100)**	**53 (53.0%)**	**45 (45.0%)**	**2 (2.0%)**
**In ddPCR panel (n = 71)**	**53 (74.6%)**	**16 (22.5%)**	**2 (2.8%)**
KRAS (n = 57)	**41 (71.9%)**	**15 (26.3%)**	**1 (1.8%)**
G12A (n = 4)	*3 (75.0%)*	*1 (25.0%)*	-
**G12C (n = 26)*	*20 (76.9%)*	*6 (23.1%)*	-
G12D (n = 12)	*9 (75.0%)*	*3 (25.0%)*	-
G12S (n = 1)	*1 (100.0%)*	*0 (0.0%)*	-
G12V (n = 8)	*5 (62.5%)*	*3 (37.5%)*	-
G13D (n = 5)	*3 (60.0%)*	*2 (40.0%)*	-
G12/G13 (n = 1)	*0 (0.0%)*	*0 (0.0%)*	*1 (100.0%)*
EGFR (n = 12)	**10 (83.3%)**	**1 (8.3%)**	**1 (8.3%)**
**Ex19Del (n = 7)*	*6 (85.7%)*	*0 (0.0%)*	*1 (14.3%)*
**G719S (n = 1)*	*1 (100.0%)*	*0 (0.0%)*	*0 (0.0%)*
**L858R (n = 2)*	*2 (100.0%)*	*0 (0.0%)*	*0 (0.0%)*
**L861Q (n = 1)*	*1 (100.0%)*	*0 (0.0%)*	*0 (0.0%)*
**S768I (n = 1)*	*0 (0.0%)*	*1 (100.0%)*	*0 (0.0%)*
BRAF (n = 2)	**2 (100.0%)**	**0 (0.0%)**	**0 (0.0%)**
**V600E (n = 2)*	*2 (100.0%)*	*0 (0.0%)*	*0 (0.0%)*
**Not in ddPCR panel (n = 29)**		**29 (100.0%)**	
KRAS	-	**8**	-
G12F	-	*1*	-
G13C	-	*4*	-
Q61L	-	*2*	-
Other	-	*1*	-
EGFR	-	**1**	-
**Ex18Del*	-	*1*	-
BRAF	-	**3**	-
K601E	-	*2*	-
D594G	-	*1*	-
MET	-	**4**	-
**Exon 14 skipping*	-	*2*	-
**Amplification*	-	*1*	-
Exon 14 (no skipping)	-	*1*	-
NRAS	-	**4**	-
Q61R	-	*2*	-
Q61K	-	*1*	-
Q61L	-	*1*	-
PIK3CA	-	**1**	-
E542K		*1*	
ALK rearrangement	-	**2**	-
TP53	-	**1**	-
IDH1	-	**1**	-
IDH2	-	**1**	-
MEK1	**-**	**1**	**-**
CTNNB1	**-**	**1**	**-**
TERT promotor	**-**	**1**	**-**

** Clinically targetable mutations*

Supplemental Table 2 – Contingency tables of mutations detected by ctDNA-ddPCR and tDNA-NGS on patient level. A) All mutations. B) Clinically targetable mutations. C) Mutations present in the ddPCR panel.

**A**		**tDNA - NGS**	
		Positive	*Negative*
**ctDNA - ddPCR**	*Positive*	53	2
	Negative	40	47

**B**		**tDNA - NGS**	
		Positive	*Negative*
**ctDNA - ddPCR**	*Positive*	32	1
	Negative	13	96

**C**		**tDNA - NGS**	
		Positive	*Negative*
**ctDNA - ddPCR**	*Positive*	53	2
	Negative	15	72

Note: The numbers represent the patients with at least one mutation detected by either ctDNA-ddPCR or tDNA-NGS. Identification of mutations by tDNA-NGS is taken as the true reference to compute the performance metrics.

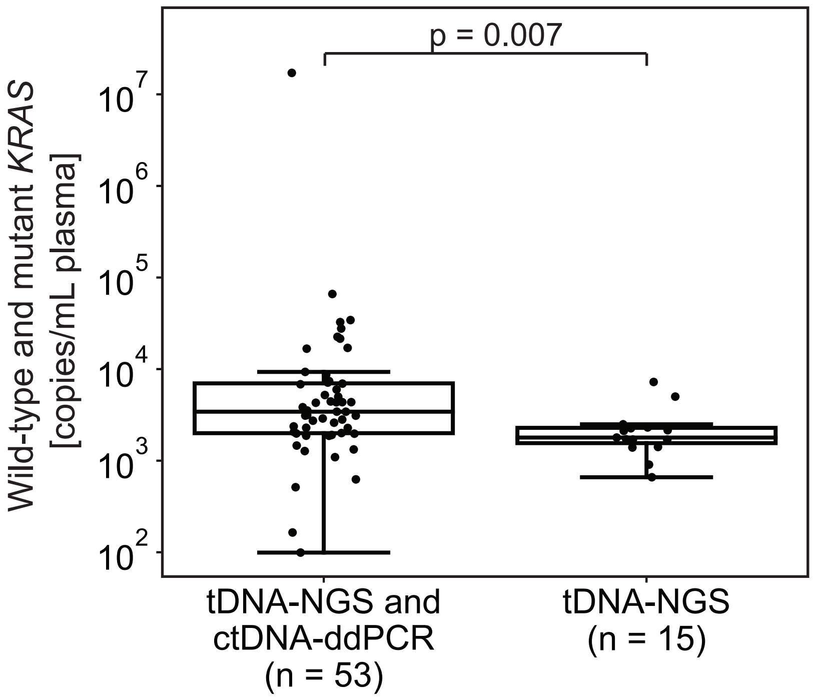

Supplemental Figure 1 - Concentrations of wild-type and mutant KRAS, reflecting the amount of cell-free DNA, in the plasma samples of patients with mutations detected by tDNA-NGS and ctDNA-ddPCR or tDNA-NGS alone. All mutations were available in the ddPCR panel.

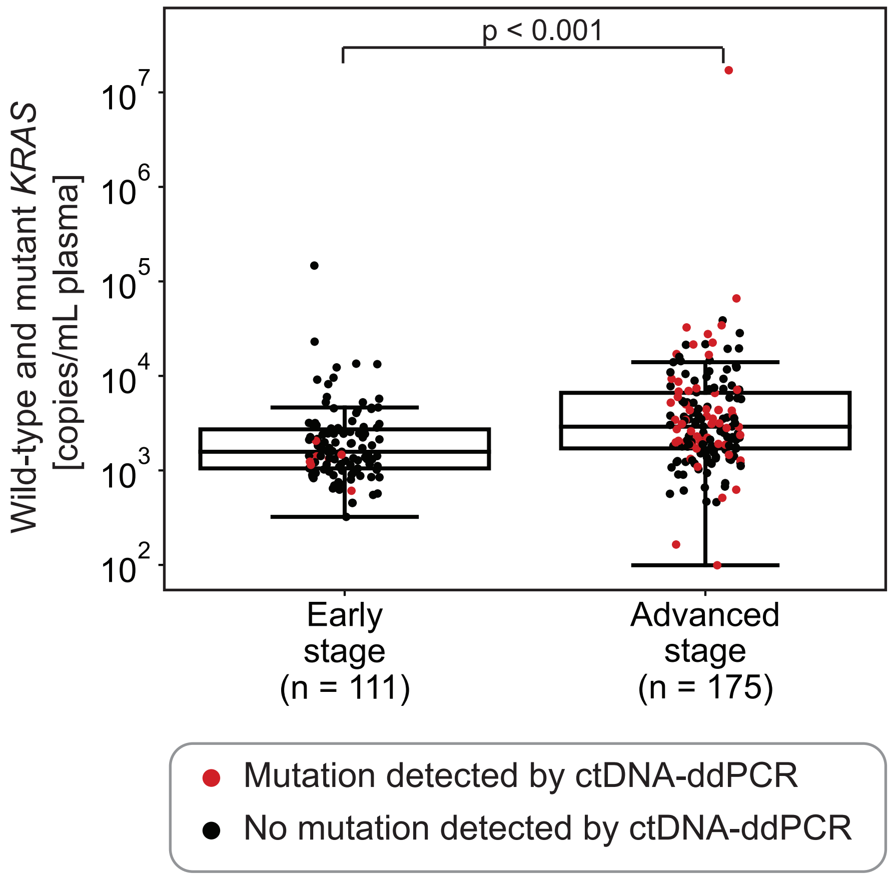

Supplemental Figure 2 - Concentrations of wild-type and mutant KRAS, reflecting the amount of cell-free DNA, in the plasma samples of early and advanced stage non-squamous NSCLC patients. The patients for whom a mutation was detected by ctDNA-ddPCR are shown in red and for whom no mutation was detected in black.
